# Sonocatalytic degradation of humic acid by N-doped TiO_2_ nano-particle in aqueous solution

**DOI:** 10.1186/s40201-016-0242-2

**Published:** 2016-01-27

**Authors:** Hossein Kamani, Simin Nasseri, Mehdi Khoobi, Ramin Nabizadeh Nodehi, Amir Hossein Mahvi

**Affiliations:** Department of Environmental Health Engineering, School of Public Health, Tehran University of Medical Sciences, Tehran, Iran; Center for Water Quality Research, Institute for Environmental Research, Tehran University of Medical Sciences, Tehran, Iran; Department of Medicinal Chemistry, Faculty of Pharmacy and Pharmaceutical Sciences Research Center, Tehran University of Medical Sciences, Tehran, Iran; Center for Solid Waste Research, Institute for Environmental Research, Tehran University of Medical Sciences, Tehran, Iran; National Institute of Health Research, Tehran University of Medical Sciences, Tehran, Iran

**Keywords:** Humic acid, *N*-doped TiO_2_, Sonocatalytic degradation

## Abstract

**Background:**

Un-doped and *N*-doped TiO_2_ nano-particles with different nitrogen contents were successfully synthesized by a simple sol–gel method, and were characterized by X-ray diffraction, field emission scanning electron microscopy, Energy dispersive X-ray analysis and UV–visible diffuse reflectance spectra techniques. Then enhancement of sonocatalytic degradation of humic acid by un-doped and N-doped TiO_2_ nano-particles in aqueous environment was investigated. The effects of various parameters such as initial concentration of humic acid, N-doping, and the degradation kinetics were investigated.

**Results:**

The results of characterization techniques affirmed that the synthesis of un-doped and *N*-doped TiO_2_ nano-particles was successful. Degradation of humic acid by using different nano-particles obeyed the first-order kinetic. Among various nano-particles, *N*-doped TiO_2_ with molar doping ratio of 6 % and band gap of 2.92 eV, exhibited the highest sonocatalytic degradation with an apparent-first-order rate constant of 1.56 × 10^-2^ min^−1^.

**Conclusions:**

The high degradation rate was associated with the lower band gap energy and well-formed anatase phase. The addition of nano-catalysts could enhance the degradation efficiency of humic acid as well as *N*-doped TiO_2_ with a molar ratio of 6 %N/Ti was found the best nano-catalyst among the investigated catalysts. The sonocatalytic degradation with nitrogen doped semiconductors could be a suitable oxidation process for removal of refractory pollutants such as humic acid from aqueous solution.

**Electronic supplementary material:**

The online version of this article (doi:10.1186/s40201-016-0242-2) contains supplementary material, which is available to authorized users.

## Background

Humic substances, as part of natural organic matters, have been a major issue in water treatment plants due to their non-biodegradability and their water-soluble formation [1, 2]. These substances can affect the water quality such as odor, taste and color. It has been also confirmed that these substances act as precursors to form disinfection by-products when water treated with chlorine [1, 3, 4]. Hence, removal of humic substances has been widely investigated for the protection of public health. In water treatment plants, portion of these substances are removed from raw water by conventional methods such as; coagulation, precipitation, filtration and adsorption [5–7]. Wang et al. reported that the removal of humic substances by using conventional processes is only 5-50 % [8].

In addition, application of high coagulant dosage isn’t reasonable due to high cost operation and problem in sludge disposal. Besides, the presence of humic substances in water may reduce the efficiency of water treatment processes when membranes or microporous adsorbents are applied.

Chemical degradation is one of the best technologies that have been widely accepted for removal of humic substances [3, 9, 10]. Recently, sonolysis process attracted considerable attention as an advanced oxidation process (AOP) for degradation of pollutants in water [11–14]. However, this method consumes considerable energy and its efficiency is low compared to other methods. In order to increase the degradation efficiency semiconductors have been added to the sonolysis processes [15, 16].

In recent years, application of heterogeneous sonocatalysis using TiO2 has become an environmentally sustainable treatment and cost-effective option for degradation of pollutants. Moreover, TiO2 is the most suitable photocatalyst for water treatment due to its high photocatalytic activity, long-time stability, relative low cost and non-toxicity [17–19]. It is well known that mechanism of sonocatalysis is similar to the photocatalysis [20, 21]. Thus, various techniques, including dye sensitization, semiconductor coupling and doping with metal and non-metal elements may enhance the sonoactivity of TiO2. According to previous studies, the doping of TiO2 with non-metal has been verified to be the most feasible method to improve photocatalytic activity of this catalyst [22]. It is also important to mention that the doping with nitrogen may be more effective than other non-metals because of its comparable atomic size with oxygen and small ionizing energy [23].

In the present study, un-doped and *N*-doped TiO_2_ nano-particles with different nitrogen contents were successfully synthesized by a simple sol–gel method and were characterized by X-ray diffraction (XRD), field emission scanning electron microscopy (FE-SEM), energy dispersive X-ray analysis (EDX) and UV–visible diffuse reflectance spectra (UV-vis DRS) techniques.

The sonocatalytic activity of the as-synthesized TiO_2_ for degradation of humic acid was investigated under ultrasonic irradiation with respect to the effects of nitrogen doping content, the initial concentration of humic acid and the addition of doped nanocatalyst into sonolysis process. Furthermore, the possible mechanism of sonocatalysis of N-doped TiO_2_ was proposed.

## Methods

### Materials

Titanium tetraisopropoxide (TTIP, Ti(OC_3_H_7_)_4_), Ethanol (EtOH), triethylamine, nitric acid (HNO_3_), Hydrochloric acid (HCl) and sodium hydroxide (NaOH) were purchased from Merck Company, Germany, as analytical grade and were used without further purifying. Humic acid was purchased from Aldrich Company as sodium salt, and it was used after preparation. The stock solution of humic acid was prepared according to the methods [24]. The humic acid solution was prepared by addition of humic acid powder into deionized water and was heated up to 60 ˚ in order to accelerate the dissolution of humic acid. Then, the humic acid suspension cooled down to room temperature and was filtered through a 0.45-μm Milipore syringe filter. The residue of humic acid on the filter was dried in an oven at 105 ˚ until stable weight. The humic acid in filtered solution was calculated by gravimetric method and stored as a stock solution for experimental use.

### Synthesis of N-doped TiO_2_

All catalyst samples were synthesized using a sol–gel method. To synthesize *N*-doped TiO_2_ with a nominal molar doping of the dopant, 3 % “TN1”, 6 % “TN2” and 12 % “TN3”, 3 mL Titanium tetraisopropoxide and a certain amount of triethylamine was dissolved in 20 mL of ethanol, and the solution was stirred for 15 min (solution A). 2 mL deionized water was added into 10 mL of ethanol that contained nitric acid, this solution was also stirred for 15 min (solution B). Solution B was added drop wise to the solution A under magnetic stirring. After constantly stirring for 30 min, the semitransparent sol was obtained. Subsequently, the obtained semitransparent sol was set for 5 h at room temperature and then dried at 80 °C for 24 h in an oven. The dried powder was ground and calcinated under air at 500 °C for 1 h with a heating rate of 16 °C min^−1^. For comparison, un-doped TiO_2_ was also synthesized without the addition of dopant under the same conditions.

### Characterization of N-doped TiO_2_

In order to determine the effect of *N*-doping on the nano-particle structure, the analysis by X-ray diffraction (XRD), surface morphology, elemental analysis and photo-physical properties were carried out. A Philips X’Pert X-ray Diffractometer with a diffraction angle range 2θ = 10–70° using Cu Kα radiation (λ = 1.5418A) was used to collect XRD diffractograms. The accelerating voltage and emission current were 40 kV and 30 mA, respectively. The average crystallite size was determined according to the Scherrer equation using the full-width at half-maximum (FWHM) of the (1 0 1) peak. The UV–visible diffuse reflectance spectra (UV-DRS) were recorded using a UV–vis spectrophotometer (Avaspec-2048-TEC, Avantes, Netherland) with BaSO_4_ as the reflectance standard. Then, the recorded data were converted to the absorbance units by using the Kubelka–Munk theory. The surface morphology and shape of the as-synthesized *N*-doped TiO_2_ was observed through a field emission scanning electron microscope (FE-SEM, TESCAN) by gold-coated samples. Energy dispersive X-ray analysis (EDX) in the FE-SEM was also taken for the elemental analysis of the doped samples.

### Sonocatalytic activity

Each suspension was prepared by adding 20 mg of each synthesized catalyst into a 100 mL of humic acid solution at concentrations 5, 10, and 20 mg L^−1^ in a reaction vessel. Prior to ultrasonic irradiation, the suspension was stirred using magnetic stirrer for 30 min in darkness to ensure a good dispersion and also to complete adsorption/desorption equilibrium of humic acid on the catalyst surface. All experiments were carried out in laboratory scale and in batch system. The ultrasonic irradiation was generated by an Elma ultrasonic bath (model TI-H5) which was operated at a frequency of 130 kHz and a maximum output power of 100 W. During the sonocatalytic processes, the solution temperature was maintained at 25 ± 2 °C using a water cooling system in ultrasonic bath. After the desired reaction time, 5 mL aliquots were withdrawn at certain interval and centrifuged at 6000 rpm for 20 min to separate the catalysts by a centrifuge (Hettich, Germany, model D-78532). The residual humic acid concentration in supernatant solution was determined by UV-vis spectrophotometer (Perkin Elmer, USA) at 254 nm. For comparison of reaction rate among different condition, the kinetic model was used.

## Results and discussion

### X-ray diffraction pattern

**Fig. 1:**
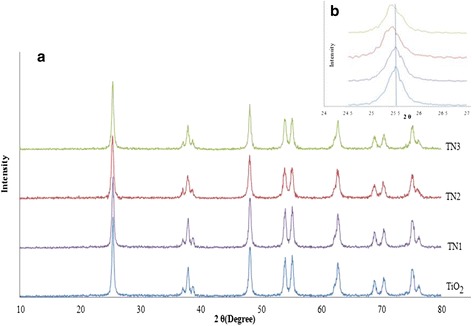
XRD patterns (**a**) and lattice distortion (**b**) of un-doped and *N*-doped nano-particles

An X-ray diffraction pattern was used to investigate the type of crystalline in material and also to know if any change was occurred after doping of TiO_2_. Figure 1a shows the XRD patterns of the un-doped and *N*-doped TiO_2_ samples. As shown in the XRD pattern, all synthesized samples had a sharp diffraction peak indicating a good characteristic crystal. The distinctive peaks at 2θ = 25.49°, 37.14°, 37.99°, 38.76°, 48.35°, 54.12°, 55.33°, 62, 90° and 68.95°; correspond to the anatase (JCPDF Card No. 20-0387) were observed. The patterns also showed that the anatase was the main phase in un-doped and *N*-doped TiO_2_ under all synthesis conditions.

These results revealed that the peak positions were nearly the same and no detectable dopant-related peaks were observed, implying that the structure of TiO_2_ has not been changed and also suggesting that nitrogen dopants do not react with TiO_2_ to form new crystalline [25, 26]. It is noteworthy, that many documents have also reported that doping with the nitrogen ions have not exhibited additional phase except anatase [22, 27]. The pure anatase phase in *N*-doped TiO_2_ could be due to the fact that the nitrogen dopants are so low and they have also moved into either the interstitial positions or into the substitution sites of the TiO_2_ crystal structure [25, 28]. Compared to the un-doped TiO_2,_ the peak of *N*-doped TiO_2_ samples exhibited a slight shift toward the lower angle corresponding to (1 0 1) plane of anatase (Fig. 1b), indicating a lattice distortion of the *N*-doped TiO_2_. These defects and disorderly state in the particles caused by nitrogen dopants are reported as key factor for absorption edge shift towards the visible-light region [25, 27].

The average crystallite size of un-doped TiO_2_ and *N*-doped TiO_2_ were calculated according to the Debye-Scherrer formula as the following:

1 $$ D=\frac{k\lambda }{\beta cos\theta} $$

where:

D = the average crystallite size,

k = a dimensionless shape factor (usually = 0.9),

λ = the wave length of the X-ray radiation (0.15418 nm for Cu Ka),

β = the full width at half-maximum of the diffraction, and

θ = the corresponding diffraction angle in degree [21].

The calculated results were 30, 30, 26 and 34 nm for un-doped TiO_2_, NT1, NT2 and NT3 nano-particles, respectively.

### FE-SEM and EDX

**Fig. 2:**
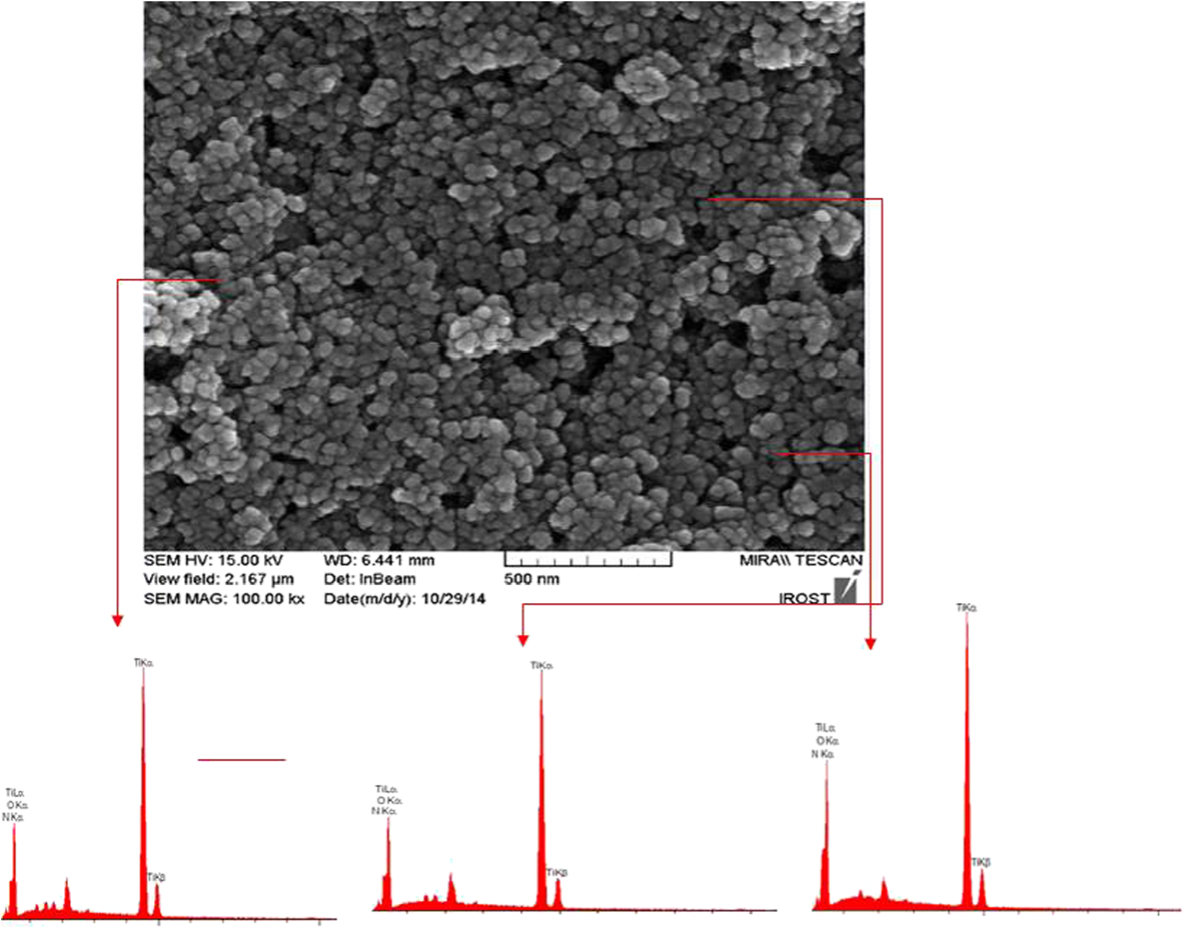
FE-SEM image and EDX spectra of *N*-doped TiO_2_ (sample TN2)

The FE-SEM was used to show the shape and morphology of un-doped and *N*-doped TiO_2_ particles (Fig. 2). The prepared nano-particles were found to be fine, irregular shape, slightly smooth surface and tend to agglomerate to form larger irregular grains. The diameter of particles was found to be 30-40 nm, which is in a good agreement with the crystal size obtained by XRD indicating that both un-doped and *N*-doped particle is nano-sized particles (Additional file 1: Figure S1). The energy dispersive X-ray Spectroscopy (EDX) of *N*-doped TiO_2_ for different points of sample shows the appearance peaks of N, O and Ti atoms, which indicating that *N*-doped TiO_2_ are mainly composed of these elements and confirm the N-doping process [29, 30]. The EDX spectra and the EDX elemental mapping (Additional file 1: Figure S2) also indicate no impurities in the samples and a good uniform distribution of N ions.

### UV-vis diffuse reflectance spectra (UV-vis-DRS)

**Fig. 3:**
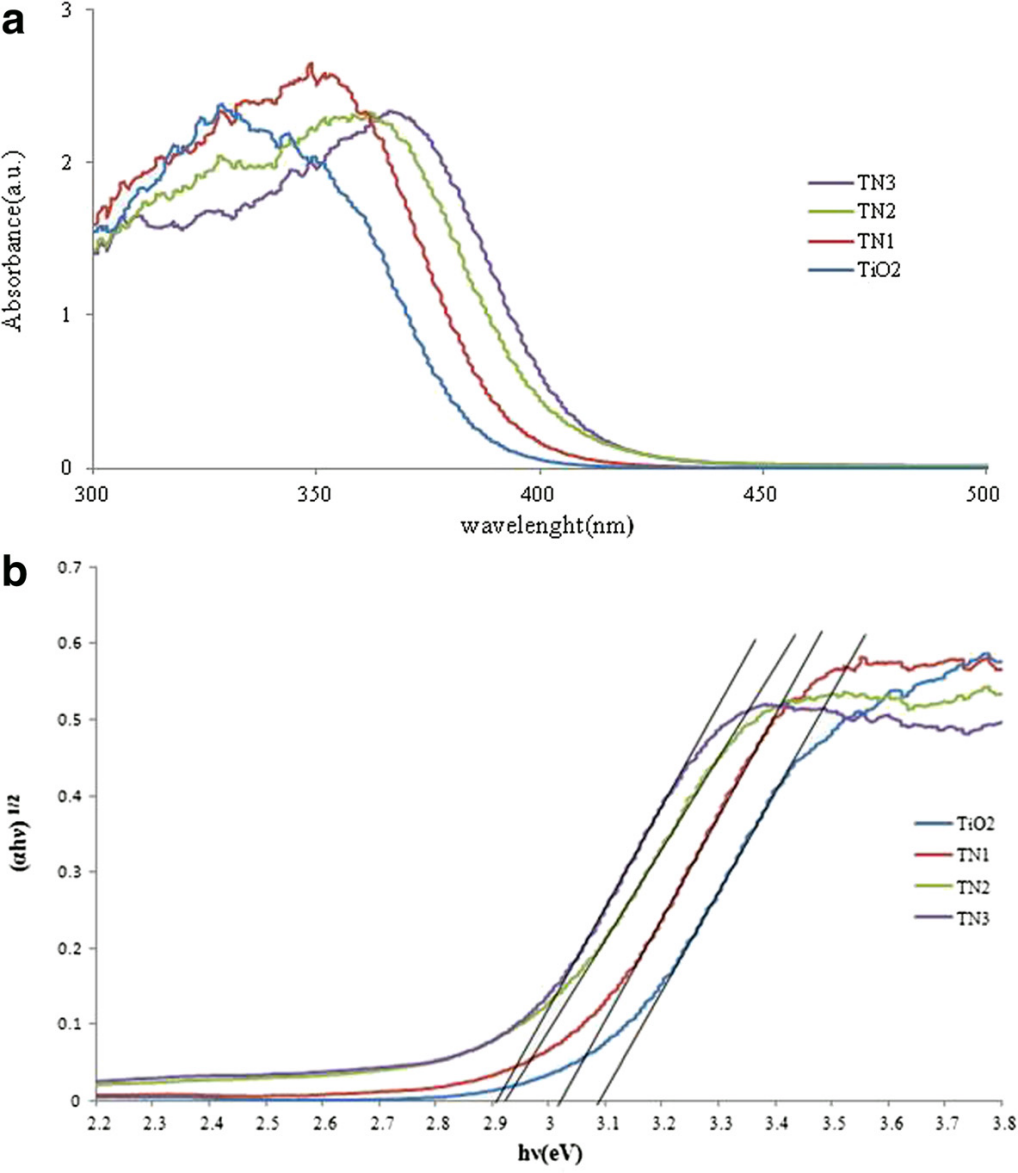
**a** UV-Vis Diffuse Reflectance Spectra and (**b**) energy band gap of un-doped and *N*-TiO_2_

UV-visible diffuse reflectance spectra are the easiest and most convenient method to have a rough measure of the influence of doping [31]. As shown in Fig. 3a, doping of TiO_2_ with nitrogen ion is clearly indicated by the reflectance spectra in the range of 300–700 nm. It is confirmed by various studies that N-doping has positive effect on the activity of the TiO_2_ photocatalyst [31, 32]. As expected, N-doping caused a red shift from UV to the visible-light region. This red shift led to a better light absorption and consequently high radical generation and degradation efficiency.

Changing toward higher light absorption and red shift of absorption edge, which is in consistent with the yellowish color of nano-particles, can be attributed to narrowing of the band gap of synthesized nano-sized particles [26].

The band gap energies (E_g_) of nano-sized particles can be determined according to the following equation [33]:

2 $$ \left(\upalpha \mathrm{h}\upnu \right)=A{\left(\mathrm{h}\upnu -{\mathrm{E}}_{\mathrm{g}}\right)}^{\mathrm{r}} $$

where α is the absorption coefficient, h is Planck’s constant, ν is the frequency of light, A is the absorption constant, E_g_ is the optical energy gap of the nano-sized particle and r is a number for characterizing the transition process, which is equal to 2 for indirect transition and 0.5 for direct transition. Therefore, the band gap energy of un-doped and *N*-doped TiO_2_ can be determined from plots of the square root of (αhν)^0.5^ versus photon energy (Fig. 3 b).

The calculated optical band gaps were 3.02, 2.92, 2.91 and 3.09 eV for the TN1, TN2, TN3 and un-doped TiO_2_ nano-particles, respectively. In all synthesized nano-particles the optical band gaps were lower than the band gap of commercial TiO_2_ (3.2 eV) that is reported in various literatures [34]. This narrower band gap enhances transition of electrons from the valence band to the conduction band in the doped TiO_2_ under ultrasonic irradiation and therefore it can increase sonocatalytic activities [34].

The decrease in the band gap of *N*-doped TiO_2_ can be attributed to the localized *N* 2p states in the structure of TiO_2_ lattice in the form of substitutional and/or interstitial N states. It has been reported that substitutional N doping decreases the band gap by mixing of the O 2p and N 2p orbitals, while interstitial doping creates an additional state between the valence band and conduction band [22, 34].

### Sonocatalytic performance of various sonocatalysts

**Fig. 4:**
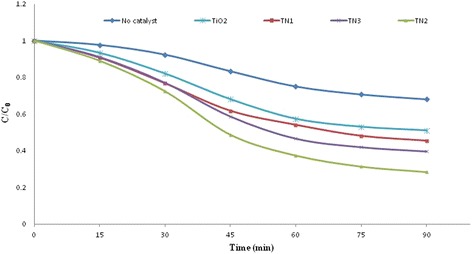
Sonocatalytic degradation of humic acid for TiO_2_ and different *N*-doped TiO_2_ nano-particles

The degradation of humic acid was studied using sonolysis, sonocatalysis with TiO_2_, and sonocatalysis with different nitrogen contents doped in TiO_2_. Figure 4 shows the degradation of humic acid under using different sonocatalysts at the neutral pH. The amount of adsorption for humic acid on the surface of the nano-particles was less than 3 % in darkness without ultrasonic irradiation; therefore it was negligible for un-doped and N-doped TiO_2_.

As shown in Fig. 4, only 32 % of humic acid was degraded under ultrasonic irradiation after 90 min (without sonocatalyst), while the degradation efficiency of TiO_2_, TN1, TN2 and TN3 sonocatalysts were 49.0, 55.0, 72.0 and 60.0.%, respectively. These results indicate that presence of sonocatalyst increases the degradation efficiency. This improvement could be due to this fact that the added sonocatalysts act as nuclei for bubble formation in aqueous solution as well as formation of oxygen vacancies in N-doped TiO_2_ crystallite [15, 21]. These oxygen vacancies act as electron-trapping sites and prevent the recombination of hole-electron pairs, while, the additional amount of surface defect such as oxygen vacancies could increase the recombination of hole-electron pairs [21, 23].

As shown in Fig. 4, the highest sonocatalytic activity was achieved by TN2 with 72.0 % for humic acid degradation after 90 min of ultrasonic irradiation. According to the reported studies, the sonocatalytic activity of doped TiO_2_ under ultrasonic irradiation is affected by different parameters such as surface area, phase of catalyst, oxygen vacancies, crystalinity of nano-particles and band gap energy [21, 23]. Therefore, the high sonocatalytic activity of TN2 could be attributed to the band gap narrowing resulting from doping of nitrogen and well-formed anatase phase. Figure 4 also indicates that the sonocatalytic activity of *N*-doped TiO_2_ initially increased with the increase of N dopant but further increasing of dopant decreased the activity. Therefore for improvement of sonocatalytic activity of TiO_2_, optimum amount of dopant is essential.

### Kinetic study

The sonocatalytic degradation of humic acid can be well explained by a pseudo-first-order reaction and its kinetics can be expressed with the following equation:

3 $$ \ln \left(\frac{{\mathrm{C}}_0}{\mathrm{C}}\right)={\mathrm{k}}_{\mathrm{app}}\mathrm{t} $$

where *k*_*obs*_ is the apparent reaction rate constant, C_0_ and C are the humic acid concentrations at initial and at time t, respectively. The *k*_*obs*_ were determined from the slopes of straight lines obtained by plotting *ln(C*_*0*_*/C)* versus irradiation time.

The values of apparent reaction rate constants (*k*_*app*_) related to the various synthesized nano-sized particles are presented in Table 1. The correlation coefficients above 0.98 indicated the sonodegradation of humic acid by un-doped and *N*-doped TiO_2_ suspensions obey the first-order kinetic model in solution. These results also indicated that reaction rate of humic acid can be improved by doping of nitrogen into the TiO_2_ structure. The apparent reaction rate constant of un-doped TiO_2_ was 0.84 × 10^-2^ min^-1^, while the apparent reaction rate constant of TN2 was 1.56 × 10^-2^ min^-1^. In addition, enhancement of the reaction rate constants of TN1, TN2 and TN3 were 1.98, 3.25 and 2.40 times higher than the reaction rate constant of sonolysis without catalyst, respectively. These results are in accordance with the those reported by Huang et al. [35] and Wu et al. [33] who studied the degradation of organic pollutants by un-doped TiO_2_ and ion- doped TiO_2_.

**Table 1:** Results of kinetic constant, k_app_, relative increase and removal efficiency of different *N*-doped TiO_2_

Catalyst	k_app_.10^-2^(min^-1^)	Relative increase	R^2^	Removal efficiency after 90 min
Absent of catalyst	0.48	1.00	0.9868	32.0
TiO_2_	0.84	1.75	0.9851	49.0
TN1	0.95	1.98	0.9895	55.0
TN2	1. 56	3.25	0.9869	72.0
TN3	1. 15	2.40	0.9846	60.0

### Effect of initial humic acid concentration

**Fig. 5:**
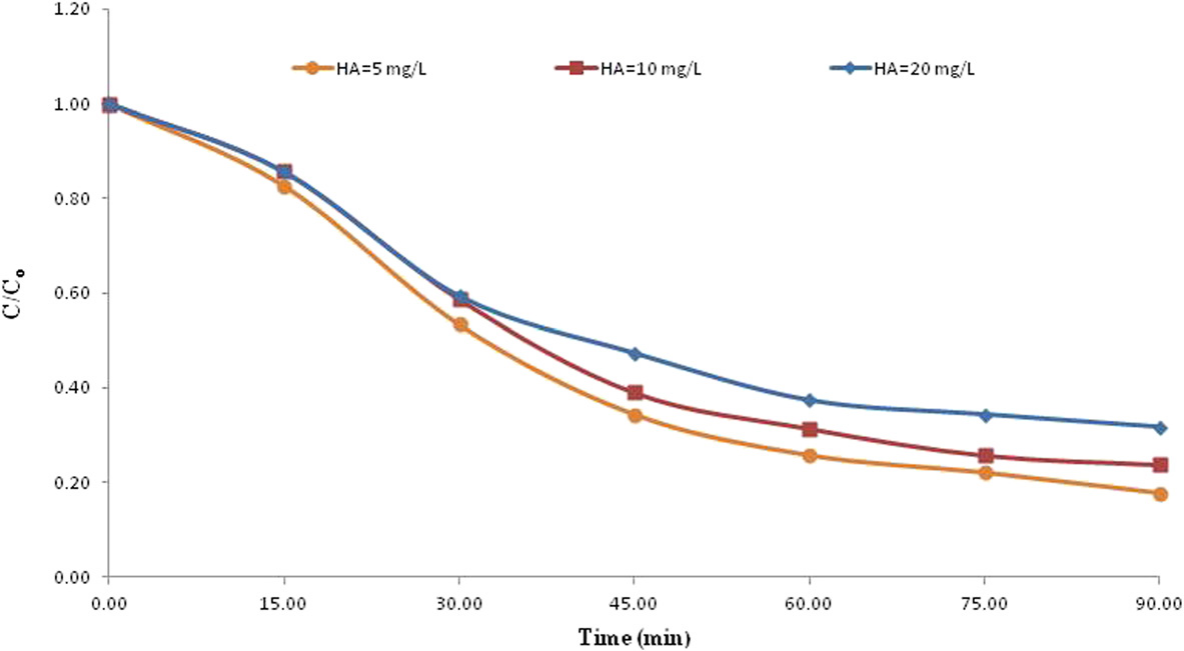
Effect of initial humic acid concentration on sonocatalytic degradation of humic acid by *N*-doped TiO_2_ (TN2) (catalyst concentration: 100 mg L^-1^)

The initial concentration of solute in aqueous environment is a key factor on sonocatalytic degradation. As shown in Fig. 5, the degradation efficiency of humic acid increased with decrease in its initial concentration. Sonocatalytic degradation of humic acid with the initial concentrations of 5, 10, and 20 mg L^-1^ for 90 min lead to the conversion of 82.0, 76.0 and 68.0 % of humic acid, respectively. This result indicates that the high degradation efficiency could be obtained at lower humic acid concentration. Our results are in good agreement with the results reported in literature [36]. This result can be due to this fact that under the same conditions, the amount of formed radicals during the sonocatalytic reaction was equal in the entire volume of the solution; therefore, the reaction of humic acid molecules with radicals becomes more likely at lower humic acid concentrations [15].

Langmuir–Hinshelwood model is widely used for analysis of heterogeneous sonocatalytic degradation kinetics as well as to realize the dependence of observed initial reaction rate on the initial concentration of solute in the aqueous environment [9, 29, 37, 38]. The L-H kinetic model is defined as the following equation:

4 $$ \mathrm{r}=-\frac{\mathrm{dc}}{\mathrm{dt}}={\mathrm{k}}_{\mathrm{r}}{\uptheta}_{\mathrm{x}}=\frac{{\mathrm{k}}_{\mathrm{r}}\mathrm{K}\mathrm{C}}{1+\mathrm{K}\mathrm{C}} $$

where r is the reaction rate (mg L^-1^ min^-1^), C is the concentration of solute at any time (mg L^-1^), t is the reaction time (min), k_r_ is the Langmuir-Hinshelwood reaction rate constant, related to the limiting rate of reaction at maximum coverage for the experimental condition (mg L^-1^ min^-1^) and K is the Langmuir adsorption constant reflecting the proportion of solute molecules which adhere to the catalyst surface (L mg^-1^) and θ is the fraction of the surface of TiO_2_ covered by solute. A linear expression of L-H model can be obtained by linearzing the Eq. (4) as follows:

5 $$ \frac{1}{r_0}=\frac{1}{k_r}+\frac{1}{k_rK{C}_0} $$

**Fig. 6:**
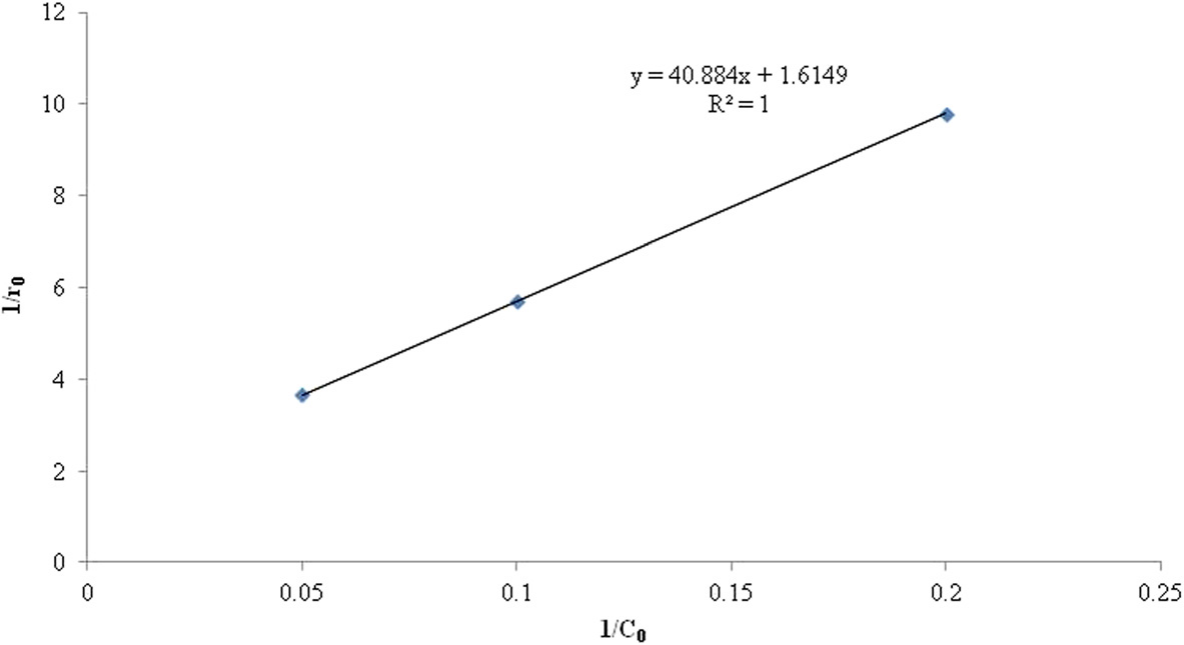
Variation of reciprocal initial rate versus the reciprocal initial concentration of humic acid

The parameters k_r_ and K which were calculated by plotting the reciprocal initial rate against the reciprocal initial concentration were 0.62 mg L^-1^ min^-1^ and 0.04 L mg^-1^, respectively (Fig. 6). As shown in Fig. 6, from the correlation coefficient above 0.98 it could be observed that the experimental data are in good agreement with L-H model. According to the L-H model, the reaction is first order at low concentration and zero order at high concentration.

### Possible mechanism

In sonolysis process, the sono-luminescence and localized hot-spots with high temperatures up to 5000 K and high pressures (approximately1800 atm) caused by acoustic cavitation and collapse of micro-scale bubbles will occur [11, 12, 39]. These hot spots can pyrolysis water molecules to OH^′^ and H^′^ radicals as below Eq. (6):

6 $$ {H}_2O + \left)\right)\Big)\to O{H}^{\kern0.5em \hbox{'}}+{H}^{\kern0.5em \hbox{'}} $$

In addition, the sono-luminescene could induce the formation of flash light/energy which equals or exceeds the band gap energy of TiO_2_ to excite the all synthesized nano-sized particles. The electron excitation from the local state of N 2p result in the generation of conduction band electrons (e−) and valence band holes (h^+)^ as shown by Eqs. (7) and (8):

7 $$ \left)\right)\Big)\to \mathrm{light}\ \mathrm{or}\ \mathrm{energy} $$

8 $$ \mathrm{N}\hbox{-} \mathrm{doped}\hbox{-} {\mathrm{TiO}}_2 + \left)\right)\Big)\to {\mathrm{h}}^{+}+{\mathrm{e}}^{-} $$

These charges migrate to the surface and finally react with a suitable electron donor and acceptor. The electrons are captured by Ti^4+^ to form Ti^3+^ states. Subsequently, the 3d orbital of Ti^3+^ ions are localized at 0.75–1.18 eV below the bottom of the conduction band. Ti^3+^ is known to be the most reactive site for oxidation process because it may cause more oxygen vacancy sites, as well as oxygen molecule is more easily adsorbed on TiO_2_ surface. Besides, the electrons will react with these surface adsorbed oxygen molecules (O_2_) to form superoxide radical anion (O_2_^′^) (Eq. 3) and is transformed further to hydroxyl radical (OH^′^) as shown in Eqs. (9) – (14).

9 $$ {e}^{-}+T{i}^{4+}\to T{i}^{3+} $$

10 $$ {e}^{-}+{O}_{2(ads)}\kern0.5em \to {O}_2^{\kern0.5em \hbox{'} - } $$

11 $$ 2{O}_2^{\hbox{'}-}+2{H}_2O\to 2{H}_2{O}_2+{O}_2 $$

12 $$ {O}_2^{\hbox{'}-}+{H}^{+}\to HO{O}^{\kern0.5em \hbox{'}} $$

13 $$ HO{O}^{\kern0.5em \hbox{'}}+{H}_2O\to {H}_2{O}_2+O{H}^{\kern0.5em \hbox{'}} $$

14 $$ {H}_2{O}_2 + \left)\right)\Big)\to 2O{H}^{\kern0.5em \hbox{'}} $$

The holes migrate to the surface and react with water molecules or chemisorbed OH^-^ on the surface of N–doped TiO_2_ and result in formation of OH′ radicals (Eqs. (15) and (16)). Besides, the holes can directly oxidize organic substances adsorbed on the surface of catalyst (Eq. (17))

15 $$ {h}^{+}+Ti\ \hbox{-}\ O{H}^{-}\to Ti\ \hbox{-} O{H}^{\kern0.5em \hbox{'}} $$

16 $$ {H}_2O+{h}^{+}\to O{H}^{\kern0.5em \hbox{'}}+{H}^{+} $$

17 $$ organic\  substances + {h}^{+}\to\ degraded\  products $$

where “)))” denotes to the ultrasonic irradiation. It is widely accepted that O_2_^′-^ and ^OH′^ have strong oxidative degradation potential. Wu et al. found that the amounts of the produced ^OH′^ radicals increase with doping of TiO_2_ [33] . In this study, from degradation efficiency it can be understand that the highest amount of radicals is generated on the surface of TN2 because narrower band gap of TN2 facilitates the transition of electron from the valence band to the conduction band and eventually increases sonocatalytic activity. Thus, optimum amount of nitrogen dopant play an important role in improving sonocatalytic activity.

## Conclusions

In this study, a simple sol-gel method was used to synthesize of un-doped and *N*-dope TiO_2_ for activity enhancement of sonolysis and sonocatalysis processes. The characterization of synthesized nano-particles was carried out by XRD, FE-SEM, EDX and UV-vis spectra. The characterization experiments confirmed that nitrogen doping has been successfully done in the TiO_2_ structure.

The degradation of humic acid was used to evaluate the sonocatalytic activity of synthesized nano-particles. On the basis of the above results and discussion, addition of nano-catalysts could enhance the degradation efficiency of humic acid as well as *N*-doped TiO_2_ with a molar ratio of N/Ti as 0.06 was found the best nano-catalyst among the investigated catalysts. The synthesized *N*-doped TiO_2_ showed about 1.86 times higher sonocatalytic activity for humic acid degradation compared to the un-doped TiO_2_.

The sonocatalytic degradation of humic acid with different catalysts followed the first-order kinetic model. L-H model confirmed the dependence of initial reaction rate on the initial humic acid concentrations and showed that the degradation efficiency decrease with the increase of initial humic acid concentrations. As a general conclusion, the results indicated that sonocatalytic degradation with nitrogen doped semiconductors could be a suitable oxidation process for removal of refractory pollutants such as humic acid from aqueous solution.

## Additional file

Additional file 1:
**Figure S1.** SEM image of pure TiO_2_. **Figure S2.** EDX elemental mapping of N-doped TiO_2. (DOC 627 kb)_
