# Supplementary material for: Sonocatalytic degradation of humic acid by N-doped TiO2 nano-particle in aqueous solution
Source: J Environ Health Sci Eng. 2016 Jan 27;14:3. doi: 10.1186/s40201-016-0242-2 (PMC4729171; doi:10.1186/s40201-016-0242-2)
Supplement: Additional file 1: — Figure S1. SEM image of pure TiO2. Figure S2. EDX elemental mapping of N-doped TiO2. (DOC 627 kb) [file 40201_2016_242_MOESM1_ESM.doc]

**Supplementary Material**

**Sonocatalytic degradation** **of humic acid by N-doped TiO_2_ nano-particle in aqueous solution**

Hossein Kamani ^1^, Simin Nasseri ^1,2^, Mehdi Khoobi ^3^, Ramin Nabizadeh Nodehi ^1^, Amir Hossein Mahvi ^1^, ^4^, ^5^*

^1^ Department of Environmental Health Engineering, School of Public Health, Tehran University of Medical Sciences, Tehran, Iran

^2^Center for Water Quality Research, Institute for Environmental Research, Tehran University of Medical Sciences, Tehran, Iran

^3^ Department of Medicinal Chemistry, Faculty of Pharmacy and Pharmaceutical Sciences Research Center, Tehran University of Medical Sciences, Tehran, Iran

^d^ Center for Solid Waste Research, Institute for Environmental Research, Tehran University of Medical Sciences, Tehran, Iran

^e^ National Institute of Health Research, Tehran University of Medical Sciences, Tehran, Iran

*Corresponding author Email: [ahmahvi@yahoo.com](mailto:ahmahvi@yahoo.com) Tel: +989123211827 Fax: +98216662267


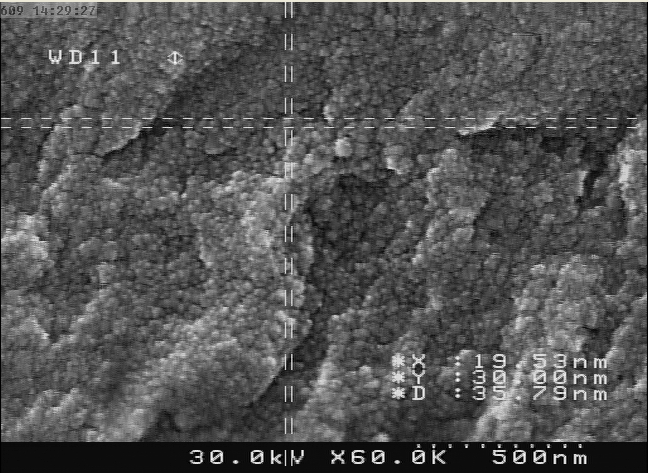


**Supplement 1**. SEM image of pure TiO_2_


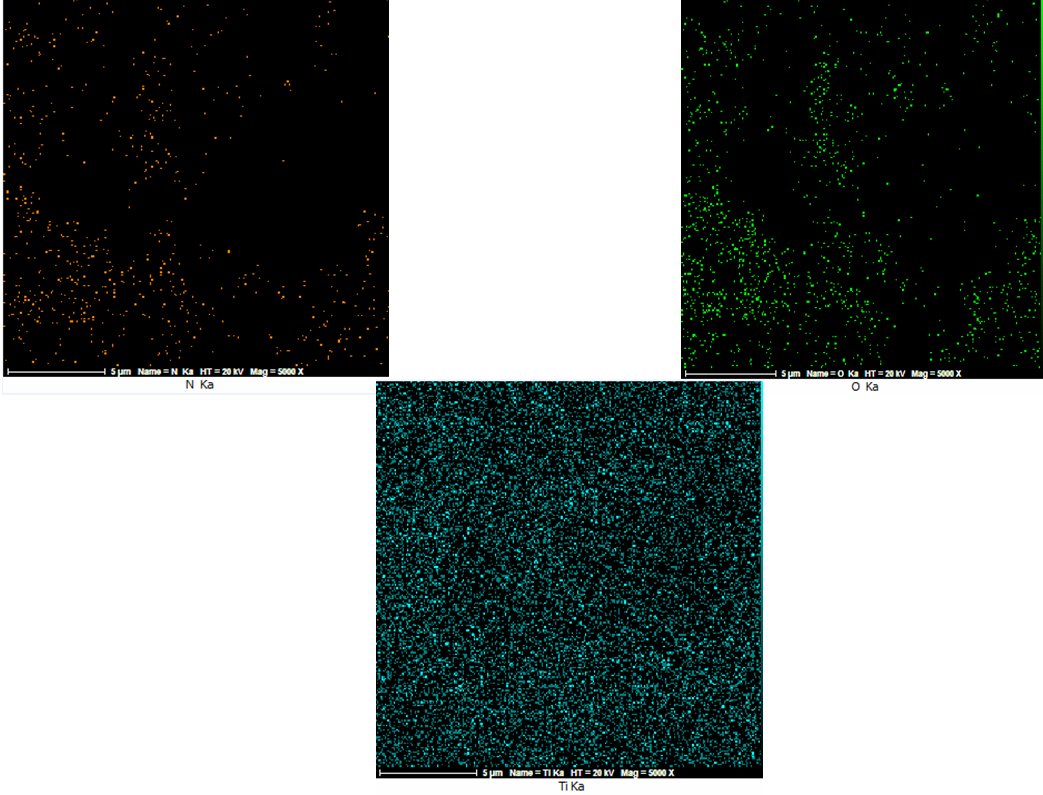


**Supplement 2**. EDX elemental mapping of N-doped TiO_2_
